# Novel methodology for construction and pruning of quasi-median networks

**DOI:** 10.1186/1471-2105-9-115

**Published:** 2008-02-25

**Authors:** Sarah C Ayling, Terence A Brown

**Affiliations:** 1 Manchester Interdisciplinary Biocentre, University of Manchester, 131 Princess Street, Manchester M1 7DN, UK

## Abstract

**Background:**

Visualising the evolutionary history of a set of sequences is a challenge for molecular phylogenetics. One approach is to use undirected graphs, such as median networks, to visualise phylogenies where reticulate relationships such as recombination or homoplasy are displayed as cycles. Median networks contain binary representations of sequences as nodes, with edges connecting those sequences differing at one character; hypothetical ancestral nodes are invoked to generate a connected network which contains all most parsimonious trees. Quasi-median networks are a generalisation of median networks which are not restricted to binary data, although phylogenetic information contained within the multistate positions can be lost during the preprocessing of data. Where the history of a set of samples contain frequent homoplasies or recombination events quasi-median networks will have a complex topology. Graph reduction or pruning methods have been used to reduce network complexity but some of these methods are inapplicable to datasets in which recombination has occurred and others are procedurally complex and/or result in disconnected networks.

**Results:**

We address the problems inherent in construction and reduction of quasi-median networks. We describe a novel method of generating quasi-median networks that uses all characters, both binary and multistate, without imposing an arbitrary ordering of the multistate partitions. We also describe a pruning mechanism which maintains at least one shortest path between observed sequences, displaying the underlying relations between all pairs of sequences while maintaining a connected graph.

**Conclusion:**

Application of this approach to 5S rDNA sequence data from sea beet produced a pruned network within which genetic isolation between populations by distance was evident, demonstrating the value of this approach for exploration of evolutionary relationships.

## Background

Phylogenies reconstructed from DNA data are usually depicted as hierarchical, bifurcating trees, but such trees are inappropriate when intraspecific phylogenies are studied because recombination between taxa, the persistence of ancestral alleles and the presence of multiple descendents from single ancestors give rise to a reticulated and multifurcating pattern of relationships [1]. Networks rather than hierarchical trees are therefore more suitable for studying intraspecific relationships [2]. Two main types of network have been used: true phylogenetic networks which aim to reconstruct the phylogenetic history of a set of sequences, and within which nodes represent ancestral sequences and edges represent evolutionary events; and character-display networks which display all conflict within the dataset, nodes not necessarily representing ancestral sequences and some edges not corresponding to true events [3]. To construct a true phylogenetic network the dataset must be relatively small and reticulations must be rare, and often a number of networks must be evaluated to identify the optimal one. Character-display methods are therefore more popular, especially when combined with pruning methods that reduce the number of nodes and edges, giving a topology that approximates with a phylogenetic network.

Various character-display methods have been used to study intraspecific phylogenies, including reticulated networks [4], statistical parsimony [5], split decomposition [6], median-joining networks [7] and Neighbor-Net [8], but median networks [9] are the most effective at displaying conflicts in the evolutionary histories of sequences while preserving the relative distances between sequences within the network. A median network or Buneman graph [10] is a network containing nodes representing binary strings and edges connecting those nodes whose strings differ by a single character. The network identifies possible ancestral sequences, depicts reticulations indicating recombination or homoplasy, and contains all the most parsimonious trees connecting the sequences from the initial alignment [9]. When median networks are constructed from DNA sequence alignments [9,11], the sequences are converted into *n *binary vectors of length *k*, *n *being the number of unique sequences in the alignment and *k *the number of unique binary positions. Constant positions are discarded and positions displaying more than two characters are either converted into sets of binary characters [9] or removed from the analysis [11], identical columns being pooled to generate a set of unique positions. The network can be thought of as being embedded in a *k*-dimensional hypercube with nodes representing sequences located at the hypercube's vertices. Two sequences are joined if they differ by one character. To achieve a connected graph, medians are generated that represent missing intermediates between observed sequences. The hypothetical median sequences may correspond to extinct ancestral sequences or existing sequences not sampled in the dataset; their nodes are called latent vertices [11]. One mechanism to generate the median network takes triplets of binary sequences, the majority character state at each position being chosen to generate the sequence which connects the three most parsimoniously. This procedure is repeated for all triplets of sequences, including newly generated medians, until no new median sequences are made; the resulting network is called the median closure.

A quasi-median network is the generalisation of a median network where characters can have more than two states. DNA sequences have five possible character states at any given position – A, T, G, C or indel (insertion/deletion). A DNA sequence alignment where only two bases are observed at each position (binary) can be represented as a string of binary characters; for these sequences the quasi-median network would coincide with the median network and could be embedded within the vertices of a hypercube. For alignments with more than two bases at any given position (multistate), the network can contain complete graphs *K*_*n*_, where *n *represents the number of character states observed at a given position. Therefore quasi-median networks containing multistate characters cannot be embedded in the vertices of a hypercube. Various approaches have been used to generate median networks for alignments containing multistate characters. These include discarding multistate characters [11], which is unsatisfactory because phylogenetic information contained within the multistate positions is lost, and a generalisation of median networks called the relation graph [12], which allows incorporation of multistate characters but which does not always give the quasi-median network and is not necessarily connected [13]. A third possibility is to convert the multistate characters into sets of binary characters by generating one character to represent the transversion (conversion between purine and pyrimidine) and one or two additional characters to represent transitions (conversion from a purine to a purine or pyrimidine to pyrimidine) [9]. The limitation with this approach is that the choice of character ordering is arbitrary: for example, a multistate position comprising A, C and T could be represented as a transversion between A and T with a transition from T to C, or a transversion between A to C and a transition from C to T. In this way the conversion of A to T can require one event or two depending on which ordering of events is chosen. The difficulties with these approaches might not be significant when a small number of closely related sequences is studied, but multistate characters become more common when larger numbers of sequences are aligned and/or more diverse sequences are compared.

An additional problem with median networks is the complexity of the topology that is obtained if there is extensive incompatibility between characters. Two characters are said to be incompatible if all combinations of the binary character states exist within the alignment; for example, if the states are '0' and '1' then any observations of '00', '01', '10' and '11' will result in a reticulation within the network. If no pairs of characters are incompatible the median network will be a tree, increasing numbers of incompatible states will introduce increasing numbers of reticulations. If all character pairs are incompatible then the median network constructed from sequences of length *n *will contain 2^*n *^nodes and will be an *n*-dimensional hypercube. In the case of quasi-median networks, multistate characters also introduce reticulations. Pairs of characters are said to be strongly compatible where for a given pair of characters, there exists a pair of character states such that no sequence is observed which does not match one of these states. Where pairs of characters are not strongly compatible the quasi-median network will contain all possible combinations of character states for these columns [14]. Hence the quasi-median closure for small sets of sequences with many character pairs which are not strongly compatible can contain large numbers of latent vertices and be too dense to visualize effectively.

Simplification of network topology can be achieved by graph reduction, in which certain characters are converted into sets of characters to reduce the number of incompatible states within the alignment, based upon how many sequences contain that character state (frequency) and how often a character is observed (weight) [9]. The reduction is based on coalescent theory and as such is not suitable for datasets in which recombination has occurred: whilst conversion of incompatible characters into sets of characters is acceptable when incompatibility has arisen because of homoplasy, recombination produces reticulations which should be retained if the network is to give a true indication of evolutionary relationships between sequences.

A second reduction method has been devised in which pruning of latent vertices is based on the properties of tuples of characters [11]. For an unobserved median to be retained, for any *k*-tuple of positions in its sequence there must be either a non-median sequence which matches all *k *positions or no non-median sequence which fails to match all of the *k *positions. When *k *= 2 this criterion merely defines a binary median sequence, in order for a sequence to be a median every pair of positions within it must have been observed in a real sequence. When *k *= 3..*n*, median sequences which contain certain *k*-tuple differences are removed from the network [11]. For quasi-median networks the inclusion of multistate characters results in vertices within the quasi-median closure which may fail to satisfy the necessary criteria when *k *= 2. It is suggested [12] that the relation graph represents the graph produced by this pruning mechanism [11] extended for use with quasi-median networks. These papers, however, do not outline an approach which could readily be employed to generate relation graphs for a set of sequences.

Both pruned median networks and the relation graph can have disconnected components where disconnectedness implies evolutionary distance [11]. A disadvantage of disconnected components is the absence of information indicating how they were once related as it is not possible to identify from which region of the full network nodes were deleted to produce the subgraphs. For small datasets, the unpruned network can be displayed for comparison, but for larger networks computational limitations may prevent visualisation of the unpruned graph. The resulting loss of information can make it impossible to identify relationships between more distantly related sequences.

This paper addresses the problems inherent in construction and reduction of quasi-median networks. We describe a novel method of generating quasi-median networks that uses all characters, both binary and multistate, without imposing an arbitrary ordering of the multistate partitions. We also describe a pruning mechanism which maintains at least one shortest path (geodesic) between observed sequences, displaying the underlying relations between all pairs of sequences while maintaining a connected graph.

## Results and discussion

### Quasi-median network construction using the virtual median

**Figure 1:**
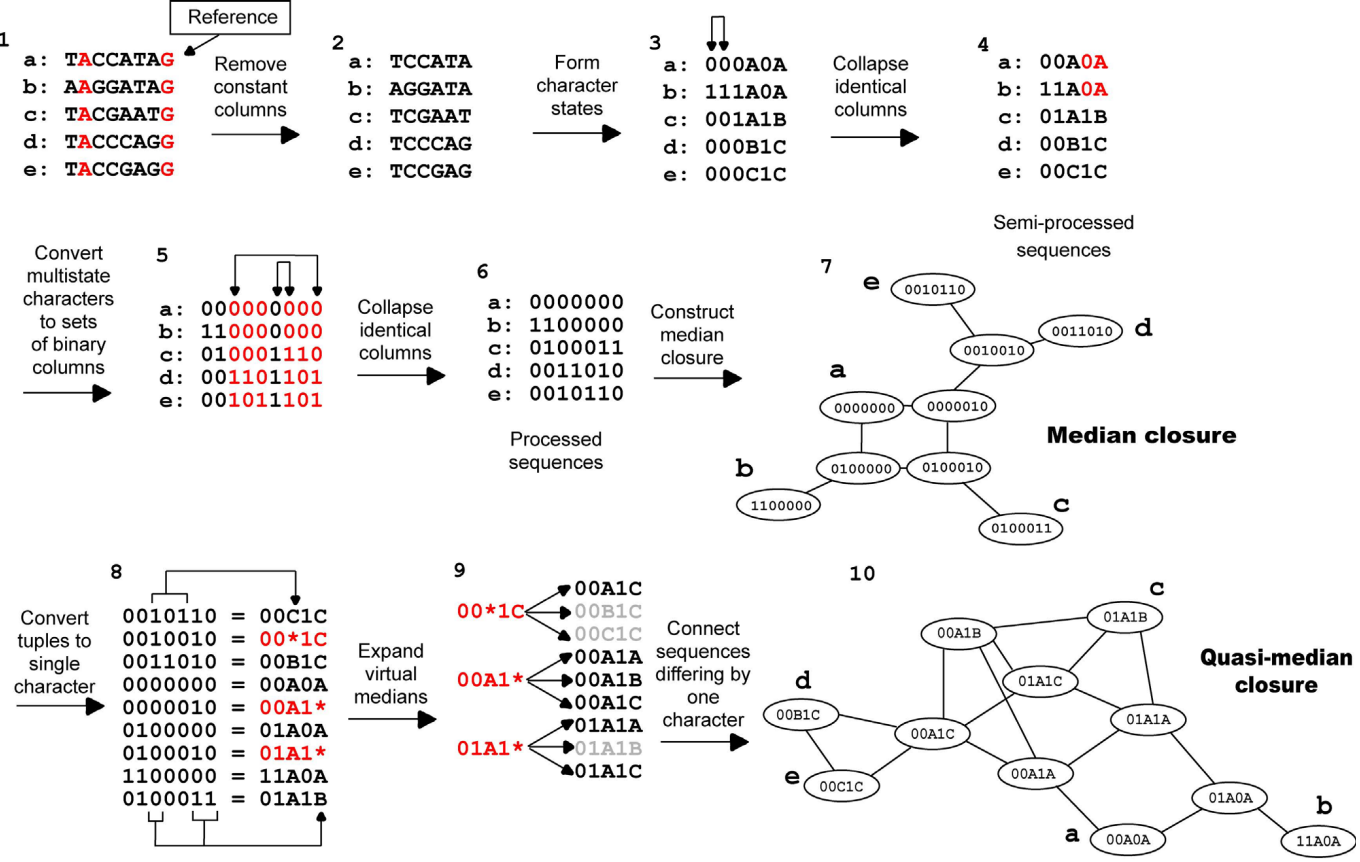
**Flow diagram illustrating quasi-median network construction for a set of hypothetical sequences**. (1) Sequence 'a' is chosen as the reference sequence, constant columns are shown in red. (2) Shows only variable positions. (3) Arrows indicate identical columns to be collapsed together. (4) In the set of semi-processed sequences the 4^th ^and 5^th ^characters contain the same partition between (0|1) and (A|BC) shown in (red|black). (5) Binary tuples representing multistate characters are shown in red. Arrows indicate identical columns to be collapsed. (6) Set of processed sequences from which the median closure (7) can be built. (8) Shows the conversion of binary tuples to multistate character states. Numbering from left to right, positions 3, 4 and 5 encode the first multistate character, 6, 7 and 3 encode the second; '*' represents the virtual median: sequences containing the virtual median are shown in red. (9) The virtual medians are expanded to form a set of sequences with each possible multistate character state. Grey sequences are those which have already been generated. (10) Shows the quasi-median closure.

Here we present a novel approach to generate quasi-median networks for a set of aligned DNA sequences. This method incorporates multistate characters by inferring virtual medians to connect them. The median closure of the sequences with virtual medians is determined, after which the virtual medians are converted to multistate characters generating the quasi-median closure (see Figure 1 for an overview). The process is outlined in detail below.

Given a multiple sequence alignment a reference sequence is chosen; the choice of sequence is arbitrary and does not alter the final network. Constant nucleotide positions within the alignment are discarded [9]. Positions containing two nucleotides are recorded in a binary format, those nucleotides which match the reference position becoming '0', others becoming '1'. For multistate positions letters are used, the reference sequence and matching bases being converted to 'A', the first alternative base to 'B' and so on. The choice of symbols to represent each character state is arbitrary, numbers have been used for binary characters and letters for multistate characters to make them more easily distinguishable for the user. After the initial encoding, identical characters are collapsed giving 'semi-processed' sequences.

**Figure 2:**
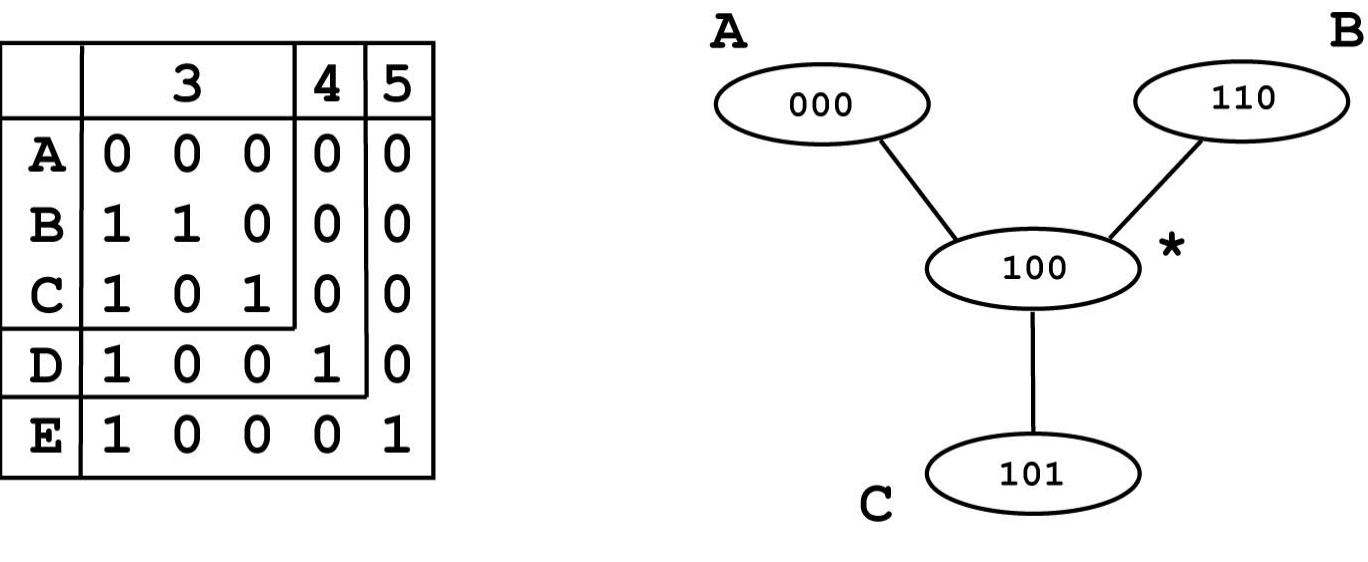
**Conversion of multistate characters to sets of binary columns**. On the left is the table used to encode each multistate character. If three character states are seen at a position the first three columns are used. There are five possible characters corresponding to the four DNA bases with a fifth character representing indels. On the right is the network constructed for three character states A, B and C connected by the virtual median '100' labelled with an asterisk.

Each character represents a partition of the data into sets, for instance those with '0' at that position and those with '1'. To generate the median closure for a set of semi-processed sequences each partition of the data must be unique [9]. Multistate characters frequently contain partitions observed in other multistate or binary characters, so the median closure cannot be constructed from the semi-processed sequences. To overcome this problem the sequences are further modified to produce binary sequences. Each multistate character is replaced with between three and five binary columns. A median network constructed for a multistate character treated in this way will be composed of nodes representing the initial nucleotides each connected to a virtual median (Figure 2). After the second round of processing, identical characters are collapsed once again, recording which characters are collapsed together. These binary sequences form the final processed sequence set. If there are no multistate characters within the initial sequence alignment the semi-processed and processed sequences are the same.

The median closure is constructed from the processed sequences as described in [9]. Each sequence generated in the median closure is then converted to the semi-processed format. To do this, each tuple of positions encoding a multistate character is examined and replaced with the corresponding character state. For tuples which encode a virtual median, a set of new median sequences corresponding to each combination of possible character states is created. The set of semi-processed sequences constitutes the nodes of the quasi-median closure, with edges connecting those nodes whose sequences differ by one character. An advantage of quasi-median networks is that the same set of sequences will always produce the same network, additionally the entire process can be automated with no parameters to be chosen by the user, ensuring consistent results and ease of use.

### Network reduction by the minimum geodesic set cover approximation

A pruned quasi-median network should accurately represent the relationships between all real sequences. One way of achieving this is to retain at least one geodesic between all pairs of observed sequences. The parsimony principle states that the simplest description of the relationship between two sequences represents a good approximation of the real evolutionary history. A geodesic is the most parsimonious means of explaining the relationship between two sequences as its length is equal to the edit distance, that is the number of character edits required to convert one semi-processed sequence into the other. Extending the parsimony principle to the entire network, an ideal method would preserve at least one geodesic between all pairs of observed sequences, such that a minimal number of latent vertices are retained in the final network.

The identification of the minimal set of latent vertices required to maintain a geodesic between all pairs of observed sequences appears to be similar to the set-covering problem [15], which is believed to be NP-hard. Whilst heuristics exist to find approximate solutions to the set-cover problem, they would require generation of the median closure which is often not feasible for networks that would typically require pruning. We therefore devised our own heuristic approach, the minimum geodesic set cover approximation (MGSCA), which uses a scoring system based on observed frequencies of character states to select a geodesic to be maintained between sequences. This can be performed for pairs of sequences and as such does not require generation of the median closure.

In MGSCA a score is assigned to each character state at every position of the semi-processed sequence alignment equal to the fraction of sequences which contain that character state at the position in question. Every node in the quasi-median closure represents a sequence. Each node is assigned a score equal to the product of the scores of the character states in its sequence. The set of geodesics is identified between each pair of observed sequences in turn and each geodesic within a set is given a score equal to the product of its nodes' scores. The highest scoring geodesic from each set is selected as it represents the pathway which is most likely given the observed set of sequences, and in the case of a tie all highest scoring geodesics are retained; any latent vertex not found on one of these geodesics is deleted from the network. The union of these highest-scoring geodesics gives the pruned quasi-median network.

**Figure 3:**
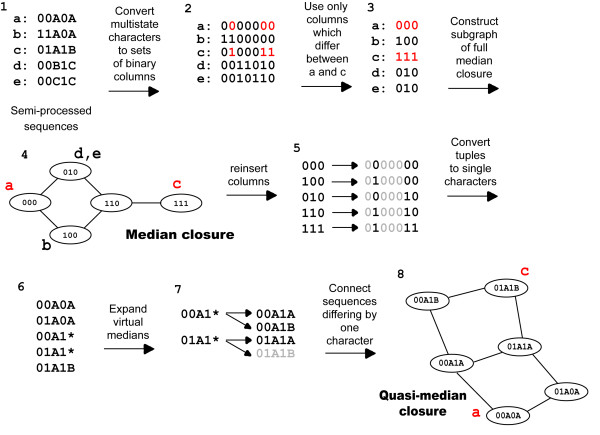
**Construction of a subgraph of the quasi-median closure for the hypothetical sequence set in Figure 1**. (1) Shows the semi-processed sequences used in Figure 1. (2) Source and destination sequences are 'a' and 'c', with characters which differ between these sequences highlighted in red. (3) Reduced set of characters from which the median closure is constructed. (4) Median closure for the reduced set of characters. (5) The full length binary sequences generated by reinserting the characters which were identical for 'a' and 'c'. (6) Binary tuples are converted to the single characters they represent; '*' represents the virtual median. (7) Virtual medians are expanded to form a set of sequences with each possible multistate character state seen in 'a' and 'c'. Grey sequences are those which have already been generated. (8) Subgraph of the quasi-median closure containing all geodesics between sequences 'a' and 'c'.

Here we present a computationally efficient approach to generate quasi-median networks. To initiate the procedure, a pair of processed observed sequences is chosen. As the generation of a quasi-median closure is independent of the order in which the characters are processed, subgraphs can be generated for each sequence pair containing only those edits required to convert one sequence to the other. An alignment is constructed from the processed sequences containing all observed sequences but only those positions where the two sequences differ – because we are interested in identifying the shortest paths between the pair, edits to any character which they have in common can be ignored. From this truncated alignment a median closure is constructed and sequences within the closure are restored to full length by addition of the characters shared by the two observed sequences. From this sequence set a quasi-median closure can be constructed as shown in Figure 1, although expansion of the virtual medians is slightly modified as only those multistate character states seen in the sequence pair need be included in the set of median sequences produced (Figure 3). This is because edits involving additional multistate character states will not occur on the shortest paths between the sequence pair. The geodesics can then be identified and assigned scores based on the frequencies of the character states at each position. The highest scoring geodesic is selected, and once the observed nodes and edges have been recorded the subgraph can be deleted, greatly reducing the size of the network required to be kept in memory. This process is repeated for all pairs of observed sequences, and the pruned quasi-median network is the union of all the selected geodesics. In median networks all nodes are connected if they differ by one character, but for visualisation purposes it is preferable to show only these high priority geodesics.

**Figure 4:**
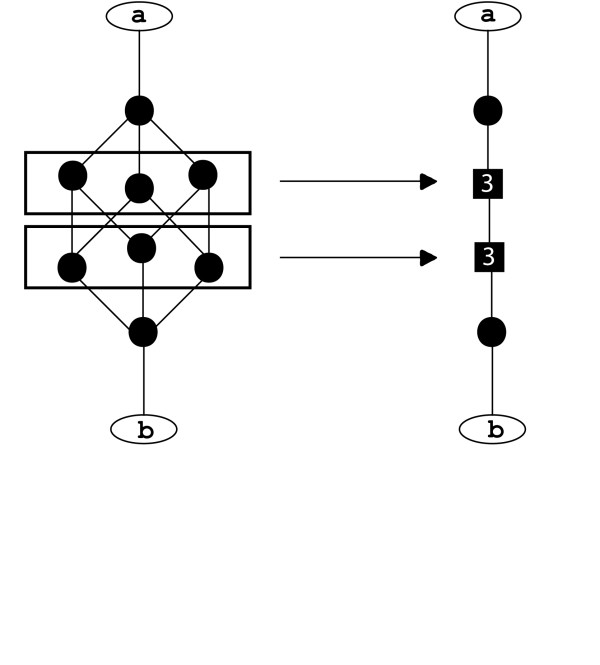
**Example showing collapse of equivalent nodes**. This example shows the collapse of two sets of equivalent nodes on the path between 'a' and 'b' into single nodes. The new nodes are shown as boxes and labeled with the number of nodes which have been collapsed.

If more than one geodesic within a set has the highest score then all equally high scoring geodesics are retained. This means that sometimes there are small sets of alternative nodes, which can be identified by observing which geodesics pass through each node. If nodes always occur at the same position on equivalent geodesics they can be collapsed into a single node to reduce network complexity, these are depicted as boxes labelled with the number of nodes that have been collapsed (Figure 4).

**Figure 5:**
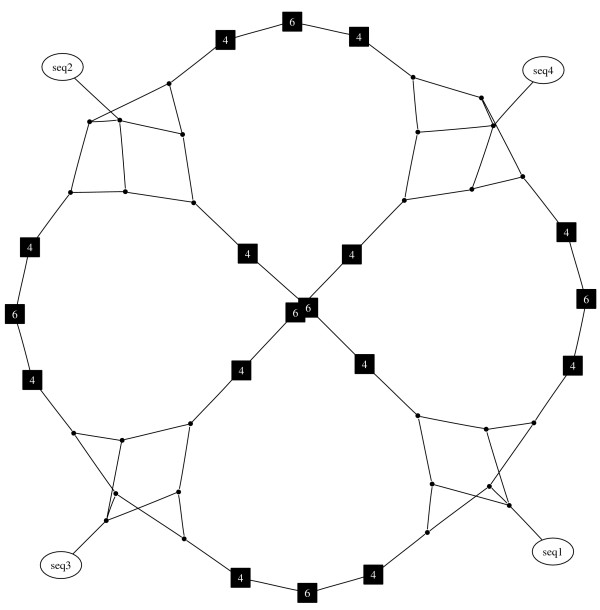
**The geodesically pruned quasi-median network for a set of four sequences displaying the maximum number of possible partitions**. Unlabelled black nodes represent single latent vertices. Labeled black nodes represent collapsed sets of equivalent latent vertices with the number displayed showing how many vertices this node represents.

**Figure 6:**
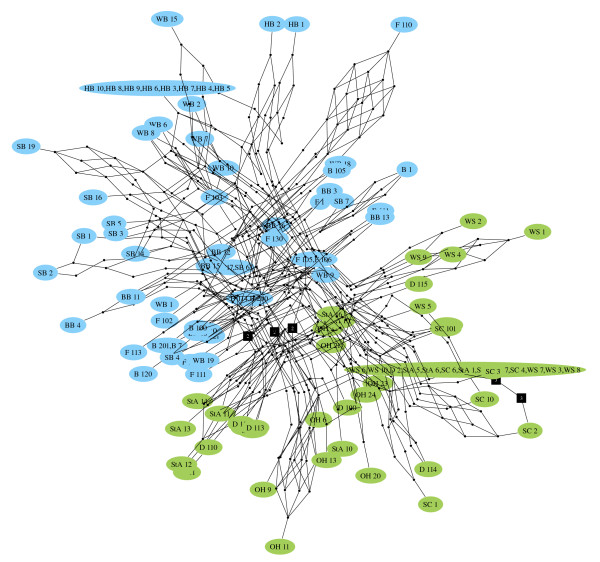
**Example of a geodesically pruned quasi-median network**. Network constructed from 110 *Beta vulgaris *ssp. *maritima *5S rDNA sequences. Cliff top populations are shown in green (locations: D, Durlston Head; OH, Old Harry; SC, Seacombe Cliff; StA, St. Aldhelm's Head; WS, Winspit) and harbour populations are shown in blue (B, Baiter; BB, Brands Bay; F, Furzey Island; HB, Holes Bay; SB, Sandbanks; WB, Wood Bar Looe). Unlabelled black nodes represent single latent vertices, labelled black nodes represent collapsed sets of equivalent latent vertices with the number displayed showing how many vertices this node represents. Edges present on high scoring geodesics are shown in black. The network shows a clear separation of the sequences from cliff top and harbour populations, in agreement with the genetic isolation displayed by these populations, as revealed by previous work with other analytical approaches [17-19].

MGSCA generates pruned quasi-median networks which are connected and which preserve the minimum path lengths between observed semi-processed sequences. Only those geodesics which are most likely given the initial set of observed sequences are retained, which greatly reduces the number of latent vertices in the network. The collapse of nodes which are always alternatives to one another allows the further reduction of the network without loss of useful information. Figure 5 shows the network constructed from four sequences displaying all possible partitions of the set of sequences as detailed in [14] from an ape mtDNA data set [16]. The initial sequences are given in Table 1A and Table 1B lists the eight virtual median sequences invoked in the generation of this network. The quasi-median closure would contain 868 nodes but the pruned quasi-median network contains a more easily viewable fifty nodes. The network shows a similar structure to the median-joining network for the same data [13], but in our network all edges are equivalent to one edit, thus maintaining a consistent representation of the pairwise distances between the sequences. When applied to the sea beet 5S rDNA data (see Methods), the procedure reduced an original quasi-median closure containing 6808 vertices to a pruned network with only 648 vertices (Figure 6). The pruned network is biologically informative as it shows a clear separation between the harbour and cliff top populations, in agreement with previous studies of these populations using isozymes, restriction fragment length polymorphisms and short tandem repeats, which have suggested that the harbour and cliff top plants display genetic isolation by distance [17-19]. Additional interpretations that can be drawn from this network regarding the evolutionary history of these populations will be described elsewhere (Ayling *et al*., in preparation).

**Table 1:** Data for the network shown in Figure 5. (A) Four sequences containing the maximum number of possible partitions. (B) Eight virtual median sequences invoked during construction of the quasi-median network for these four sequences.

A)	
seq1	0000000AAAAAAA
seq2	1100011ABBBBBB
seq3	1010101BCACCBC
seq4	1001110CCCBACD
B)	
	1000000A*A*A**
	1000001A*A**B*
	1000010A**BA**
	1000011A**B*B*
	1000100*CA*A**
	1000101*CA**B*
	1000110*C*BA**
	1000111*C*B*B*

## Conclusion

Previous studies have shown median and quasi-median networks to be useful tools for analysis of intraspecific phylogenies. We devised a new and fast method to generate quasi-median networks, using a virtual median and enabling all characters, both binary and multistate, to be used without imposing an arbitrary ordering of the multistate partitions and hence without losing phylogenetic information. We also developed a simple and intuitive pruning mechanism that reduces the number of latent vertices within a network while maintaining a connected network and preserving the geodesic lengths between all pairs of observed sequences. A great advantage of this approach is that each sequence pair can be treated separately so that the full quasi-median closure does not need to be constructed, an important consideration with relatively divergent sequences that can give rise to quasi-median networks that are too large to build. The method always produces a single network because it displays multiple equally good geodesics, therefore no arbitrary decisions have to be made during network construction, and no external parameters have to be applied. Application of this approach to 5S rDNA sequence data from sea beet produced a pruned network within which genetic isolation between populations by distance was evident, demonstrating the value of this approach for exploration of evolutionary relationships.

## Methods

The DNA sequence dataset used in this study comprised 110 sequences from the spacer regions of the 5S ribosomal DNA (rDNA) loci of sea beet (*Beta vulgaris *ssp. *maritima*). In plants, the 5S rDNA genes are arranged in tandem arrays, each gene separated by an untranscribed spacer that in sea beet is 227–230 bp in length. These spacers are among the most variable regions of plant genomes and hence are attractive markers for studying intrapopulation relationships, but their analysis by conventional tree-building is impossible because of frequent character conflict caused by recombination between spacers, and because both ancestral and derived spacer sequences are present in a single array [20]. The sequences were obtained by Dr D. Turner (University of Manchester Institute of Science and Technology, UK) and are in groups of ten sequences, each group from a different population of sea beet from the southeast coast of Dorset, UK, six populations from a harbour area and four from cliff tops. Previous studies [17-19] of isozymes, restriction fragment length polymorphisms and short tandem repeat markers have suggested that these populations display genetic isolation by distance.

## Authors' contributions

SCA carried out the development and implementation work described in this paper including writing the necessary code. TAB provided direction and aided the biological interpretation of the networks that were obtained. SCA and TAB contributed jointly to preparation of the manuscript.
